# Down-Regulation of Canonical and Up-Regulation of Non-Canonical Wnt Signalling in the Carcinogenic Process of Squamous Cell Lung Carcinoma

**DOI:** 10.1371/journal.pone.0057393

**Published:** 2013-03-07

**Authors:** Domokos Bartis, Veronika Csongei, Alexander Weich, Edit Kiss, Szilvia Barko, Tamas Kovacs, Monika Avdicevic, Vijay K. D’Souza, Judit Rapp, Krisztian Kvell, Laszlo Jakab, Miklos Nyitrai, Tamas F. Molnar, David R. Thickett, Terezia Laszlo, Judit E. Pongracz

**Affiliations:** 1 Department of Medical Biotechnology, Institute of Immunology and Biotechnology, Medical School, University of Pecs, Pecs, Hungary; 2 Department of Medicine, Medical School, University of Birmingham, Birmingham, United Kingdom; 3 Department of Biophysics, Medical School, University of Pecs, Pecs, Hungary; 4 Department of Surgery, Medical School, University of Pecs, Pecs, Hungary; 5 Department of Pathology, Medical School, University of Pecs, Pecs, Hungary; 6 Szentagothai Research Center, University of Pecs, Pecs, Hungary; H. Lee Moffitt Cancer Center & Research Institute, United States of America

## Abstract

The majority of lung cancers (LC) belong to the non-small cell lung carcinoma (NSCLC) type. The two main NSCLC sub-types, namely adenocarcinoma (AC) and squamous cell carcinoma (SCC), respond differently to therapy. Whereas the link between cigarette smoke and lung cancer risk is well established, the relevance of non-canonical Wnt pathway up-regulation detected in SCC remains poorly understood. The present study was undertaken to investigate further the molecular events in canonical and non-canonical Wnt signalling during SCC development. A total of 20 SCC and AC samples with matched non-cancerous controls were obtained after surgery. TaqMan array analysis confirmed up-regulation of non-canonical Wnt5a and Wnt11 and identified down-regulation of canonical Wnt signalling in SCC samples. The molecular changes were tested in primary small airway epithelial cells (SAEC) and various lung cancer cell lines (e.g. A549, H157, etc). Our studies identified Wnt11 and Wnt5a as regulators of cadherin expression and potentiated relocation of β-catenin to the nucleus as an important step in decreased cellular adhesion. The presented data identifies additional details in the regulation of SCC that can aid identification of therapeutic drug targets in the future.

## Introduction

Lung cancer (LC) is the leading cause of cancer death world-wide [1]. About 80% of LCs belongs to the NSCLC type which is erroneously considered as a single entity. The two main NSCLC sub-types, namely AC that arises mostly in the peripheral airways or the bronchio-alveolar region of the parenchyma, and SCC that develops mainly in the proximal airways and affects mostly cigarette smokers, differs not only in aggressiveness but responsiveness to chemotherapy, also. To make the overall picture more complicated, there are an emerging number of combined NSCLCs where malignant tumours are representing themselves as adeno-squamous or mixed type LC. Not surprisingly, the molecular background of AC and SCC development has been a focus of intense investigation. In various studies Wnt signalling has emerged as one of the potential regulators of the carcinogenic process.

### Wnt Signalling

Wnt signalling regulates a variety of developmental processes including cell fate specification, proliferation, polarity and migration (reviewed in [2]). Wnt molecules trigger gene transcription via at least three signalling pathways: the canonical or β-atenin dependent, and two non-canonical pathways. When Wnts bind to their trans-membrane receptors, Frizzleds (Fzd) and co-receptors, LRP5/6, signal transduction begins on the canonical pathway. Once stabilized, non-degraded β-catenin molecules move to the nucleus where they activate TCF-LEF-dependent gene transcription. In the absence of Wnt signals, the cytoplasmic β-catenin is subjected to phosphorylation in the APC-Axin-GSK3β-complex [2] then to subsequent proteasomal degradation. Upon non-canonical Wnt signals, the JNK/AP1 dependent, planar cell polarity (PCP) and the PKC/CAMKII/NFAT dependent Ca^2+^ pathways are activated.

### Wnt Signalling in LC

Analysis of gene expression data has revealed that Wnt pathway activity can be strongly down-regulated in small cell lung cancer (SCLC) through over-expression of inhibitory genes. Proof of deregulation of specific Wnt molecules leading to oncogenic signalling has also emerged. Frequent loss of Wnt7a mRNA was demonstrated in some studies in LC cell lines and primary tumours [3], and elevated levels of Wnt1 and Wnt2 [4], [5] have also been reported in NSCLC. NSCLC cells transformed with Wnt7a showed inhibition of anchorage independent growth [6] supporting the theory that decreased Wnt7a levels are part of the pathogenic profile of NSCLC. Furthermore, over-expression of dishevelled (Dvl), a signal transducer from Wnt receptors, Fzd-s, has been reported in 75% of NSCLC cases [7]. Down-regulation of Wnt pathway antagonists like Dkk3 [8], WIF [9], [10] and sFRP [11] has also been described. When NSCLC is concerned, the well-known epithelial-mesenchymal transformation (EMT) is a characteristic feature [12] and is generally linked to increased β-catenin dependent signalling [13]. Although β-catenin mutations in LC-s are relatively rare [14]–[16], up-regulation of uncomplexed β-catenin without genetic alteration to β-catenin itself was shown in a high proportion of human NSCLC primary tumours and tumour cell lines [17]. Recently, comparative microarray and pathway analysis of both AC and SCC samples [18], [19] have identified activation of the non-canonical Wnt signalling pathway as a main regulator of SCC development.

In the present study, the role of Wnt signalling was investigated further using primary tumours and non-cancerous lung tissues, human AC and SCC lung cancer cell lines as well as commercially available, non-cancerous, primary small airway epithelium (SAEC).

## Materials and Methods

### Ethics Statement

Lung tissue samples were collected during lung resections at the Department of Surgery, University of Pecs, Hungary. The project was approved by the Ethical Committee of the University of Pecs. Patients had given written consent to provide samples for research purposes. All collected samples were treated anonymously.

### Cell Lines

Human lung cancer cell lines A549 (AC), and H157 (SCC) were obtained from the American Type Culture Collection (Rockville, MD). Transgenic cell line Wnt11-A549 was created in our laboratory using lentiviral transgenesis [20].

### Primary Cells and Tissues

Normal small airway epithelial cells (SAEC) were commercially available (Lonza) and were isolated from lungs of multiple random donors of different sexes and ages by Lonza.

Tumour types were determined in the Department of Pathology, University of Pecs, Hungary. A total of 20 squamous and adeno lung carcinoma samples with matched normal lung samples were obtained after surgery based on the availability of frozen tissue for molecular analysis. Samples were also formalin fixed and paraffin-embedded to be sectioned at 5 µm and stained with hematoxylin and eosin (HE) for light microscopy. Following analysis, samples were grouped as SCC vs AC according to the World Health Organisation (WHO) classification [21] by a dedicated lung pathologist.

### Cell Cultures

SAEC cultures were maintained at 37°C and 5% CO_2_ content in a humidified atmosphere. For initial expansion primary SAEC-s were seeded onto 6-well plates (100 000 cells/well) and cultured in Small Airway Growth Medium (SAGM) (Lonza). For treatment SAEC-s were cultured in 24-well plates in SAGM. All other cell types (A549, H157, Wnt11-A549) were cultured in DMEM or RPMI supplemented with 10% FCS and were maintained at 37°C in 5% CO_2_ content in a humidified atmosphere.

### Recombinant Proteins, siRNA and Chemicals

Purified, recombinant Wnt11 was purchased from R&D Systems and used at two different concentrations (0.1 and 1.0 µg/ml) for treatment of cell cultures. siWnt11 and control siRNA were obtained from Invitrogen to knock down Wnt11 expression. 100 nmol negative control and Wnt11-specific Cy3 labelled siRNA oligos were transfected into target cells using Lipofectamine 2000 transfection reagent following the manufacturer’s instructions (Invitrogen). Uptake of the labelled oligonucleotides was tested using fluorescence microscopy. Cells were lysed 36 hours after transfection for RNA isolation and cDNA synthesis. qRT-PCR was used to determine Wnt11 mRNA levels in transfected samples. β-catenin inhibitor IWR-1 was purchased from Sigma, dissolved in DMSO and used at the final concentration of 1.0 µg/ml.

### Immunohistochemistry and Immunofluorescent Staining

Sections of primary lung tissues were permeabilized in PBS buffer containing 0.1% saponine and 5% bovine serum albumin for 30 minutes then incubated in primary anti-Wnt11 (AbCam), then in anti-rabbit-HRP secondary antibodies for 1 hour each. To ensure the comparability of expression levels, all images were captured with the same exposition settings. For β-catenin staining of SAEC, normal A549, Wnt11-A549 and H157 cells: 10000 cells/cm^2^ were seeded and cultured for 24 h on 4-well culture slides (BD-Falcon). The monolayer cell cultures were fixed with 4% formaldehyde and permeabilized with PBS containing 0.1% Triton-X and 5% BSA. Murine anti-β-catenin IgG_1_ mAb (Santa Cruz) (1∶50) and donkey anti-murine IgG secondary antibody conjugated to NorthernLight 557 (R&D Systems) (1∶200) were used for immunofluorescent labelling; nuclei were counterstained with DAPI. Images were acquired using an Olympus IX-81 light and fluorescent or a confocal microscope, then densitometry was performed.

### RNA Isolation, Preparation of cDNA, TaqMan Array and quantitative RT-PCR

Total RNA was prepared from cell cultures and primary lung resections (SCC and AC samples and their respective non-cancerous controls) using NucleoSpin RNAII kit (Macherey-Nagel) with on-column DNase digestion. cDNA was prepared from RNA samples with MMuLV reverse transcriptase kit (Thermo Scientific). Real-time quantitative RT-PCR examinations were carried out using ABsolute QPCR SYBR Green Low ROX master mix (ABGene) and an Applied Biosystems 7500 thermal cycler system. The sequences and data of primers are listed in Table 1.

**Table 1:** List of gene specific primers.

Gene	Accession number	Forward primer	Reverse primer	Product
**β-actin(1)**	NM_001101	5′-CTGTGCTATCCCTGTACGCCTCTG-3′	5′-GTGATCTCCTTCTGCATCCTGTCG-3′	541 bp
**β-actin(2)**	NM_001101	5′-GCGCGGCTACAGCTTCA-3′	5′-CTTAATGTCACGCACGATTTCC-3′	55 bp
**E-cad**	NM_004360	5′-GACCGGTGCAATCTTCAAA-3′	5′-TTGACGCCGAGAGCTACAC-3′	93 bp
**N-cad**	NM_002961	5′-AGCTTCTCACGGCATACACC-3′	5′-GTGCATGAAGGACAGCCTCT-3′	133 bp
**S100A4**	NM_002961	5′-TGGAGAAGGCCCTG-3′	5′-CCCTCTTTGCCCGAGTACTTG-3′	58 bp
**aSMA**	NM_001613.2	5′-CCGACCGAATGCAGAAGGA-3′	5′-ACAGAGTATTTGCGCTCCGAA-3′	88 bp
**VIM**	NM_003380	5′-ATTCCACTTTGCGTTCAAGG-3′	5′-CTTCAGAGAGAGGAAGCCGA-3′	97 bp
**Wnt5a**	NM_003392	5′-TGGCTTTGGCCATATTTTTC-3′	5′-CCGATGTACTGCATGTGGTC-3′	199 bp
**Wnt11**	NM_004626	5′-CGTTGGATGTCTTGTTGCAC-3′	5′-TGACCTCAAGACCCGATACC-3′	209 bp

Quantitative real-time RT-PCR data were analyzed by delta Ct (dCt) and Relative Quantity (RQ) methods as suggested by Applied Biosystems using the 7500 System SDS Software. Briefly, Ct values were determined for each sample using an automatic threshold level determined by the 7500 System SDS Software. Delta Ct (dCt) values were determined according to the following formula: dCt(target gene) = Ct(target gene) – Ct(housekeeping gene). Changes in gene expression are shown as RQ values calculated using the formula below: RQ = 2^−ddCt^, where ddCt values were calculated as ddCt = dCt(sample) – dCt(reference sample).

TaqMan®Array Human WNT Pathway 96-well Plate (4414100)–the gold standard of specificity and sensitivity in real-time PCR was purchased from Applied Biosystems. The array also contained 4 housekeeping genes including 18S, β-actin (ACTB), Glyceraldehyde 3-phosphate dehydrogenase (GAPDH) and hypoxanthine phosphoribosyltransferase (HPRT).

### Matrigel Invasion Assay

A549, H157, SAEC, rWnt5a (0.1 and 1.0 µg/ml) and rWnt11 (0.1 and 1.0 µg/ml)-treated SAEC were used in the invasion assays. SAEC were pre-treated with rWnt5a and rWnt11 for 72 hours before tested in the invasion assay. Cells were cultured in DMEM/RPMI/SAGM, respectively supplemented with 10% FCS and were maintained at 37°C in 5% CO_2_ content in a humidified atmosphere. Invasion assays were carried out in 24-well Matrigel invasion chambers (BD BioCoat Matrigel) with 8.0-µm filter membranes. After rehydration of the chambers, membranes were transferred to the wells containing 750 µl of DMEM/RPMI/SAGM supplemented with 10% FCS as a chemoattractant. Cells (5×10^4^) in 500 µl of FCS- or growth-factor-free DMEM/RPMI/SAGM were added into each of the chambers. After 24 hours incubation the non-invading cells on the upper side of the chamber membranes were removed. The invading cells to the opposite side of the chamber membranes were fixed in 4% paraformaldehyde and were stained using hematoxilin-eosin. The total number of invading cells was determined by counting the number of cells that migrated to the lower surface of the filters. Five separate microscopic fields were counted on each membrane.

### Statistical Analysis

Where it is applicable, data are presented as mean +/− standard deviation (SD), and the effects between various experimental groups were compared with the Student t-test. p<0.05 was considered as significant.

## Results

### Wnt Signalling Molecules are Differentially Expressed in AC and SCC

Using a commercially available Wnt Taqman array system, differences between canonical and non-canonical Wnt pathway activities were studied in AC and SCC samples, respectively. Sample pairs included pooled cDNA of primary AC (12) or SCC (8) samples and their respective tissue autologous, non-cancerous controls. The read-outs were matched to all four housekeeping genes of the array to focus on the largest of differences in gene expression.

**Figure 1:**
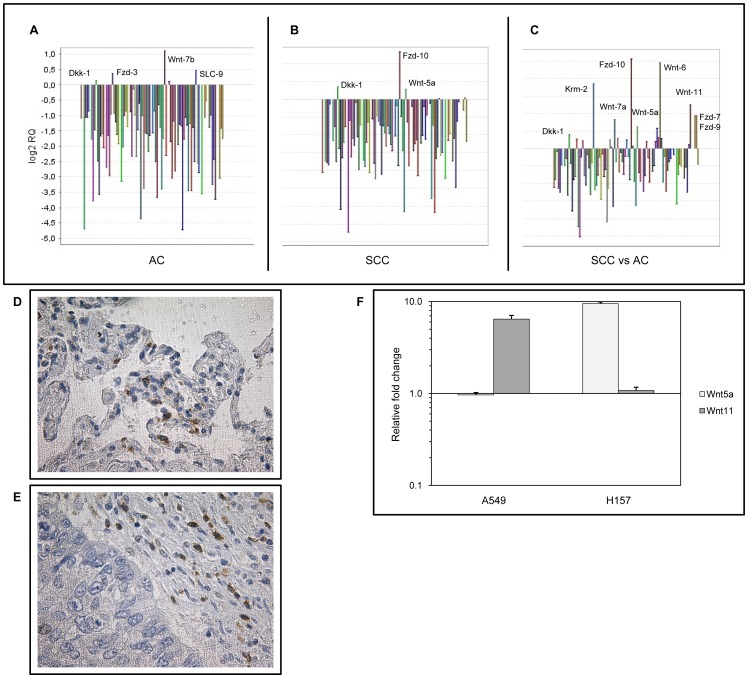
Level of Wnt signalling molecules in AC and SCC. Pooled cDNA of 12 AC, 8 SSC samples were targeted to gene expression analysis using a commercially available Taqman array. Four housekeeping genes were used (18S, GAPDH, HPRT1, GUSB). **A:** Expression profile of AC. Pooled cDNA of autologous normal tissue samples of the same AC patients served as reference. Note the increased level of the canonical Wnt-7b, and the receptor Fzd-3. (For the list of all gene expression changes see Table S1). **B:** Gene expression levels of SCC. Pooled cDNA of autologous normal tissue samples of the same SCC patients served as reference. Note the upregulation of the non-canonical Wnt5a and the canonical pathway inhibitor Dkk-1, along with increased level of Fzd-10 gene expression. (For the list of all gene expression changes see Table S2). **C:** Gene expression of SCC compared to AC. Note the increased level of non-canonical Wnts (Wnt5a and Wnt11), several receptors (Fzd-7, -9, -10), a canonical pathway inhibitor (Dkk-1) and an inhibitory receptor (Krm2). (For the list of all gene expression changes see Table S3). **D and E:** Immunohistochemical staining of primary control (Panel D) and AC (Panel E) tissues for Wnt11. Note the higher Wnt11 expression in the tumours emphasizing the relative nature of the initially identified differences at mRNA level. Images shown are representatives of three independent stainings. **F:** Wnt11 gene transcription was also measured in an AC (A549) and an SCC (H157) cancer cell line. Note the higher Wnt11 levels in the observed cancer cell lines compared to the normal, non-cancerous pulmonary epithelium (SAEC). The AC cell line showed a more pronounced increase in Wnt11 expression than the SCC cell line. (The results are representative of three independent experiments where the non-cancerous control (SAEC) was derived from three individual donors of different ages).

Increased transcription of the canonical Wnt-7b ligand and a Wnt receptor Fzd-3 was detected in pulmonary AC-s along with drastic down-regulation of canonical pathway inhibitors (Figure 1A). In contrast, activation of the non-canonical Wnt5a and canonical pathway inhibitor Dkk-1 along with up-regulation of Fzd-10 gene transcripts were measured in SCC samples (Figure 1B).

When gene expression profiles of non-cancerous control tissues of the two different tumour types were compared, no characteristic genes or molecular patterns were identified as potential initiators of AC or SCC development (data not shown).

However, comparative analysis of AC and SCC tumour samples emphasized additional differences between the two NSCLC subtypes. Much higher levels of an additional non-canonical Wnt, Wnt11, several receptors (Fzd-7, -9, Fzd-10) as well as increased levels of canonical pathway inhibitor Dkk-1 and inhibitory receptor Kremen (Krm2) were detected (Figure 1C) in SCC.

Although up-regulation of the non-canonical Wnt pathway has been identified in previous studies as a general trend in gene expression alterations during SCC development, the precise role for specific molecules in the carcinogenic process has rarely been defined. One of the enigmatic molecules in the carcinogenic process of SCC is Wnt11. As Wnt11 up-regulation in SCC was detected only as relative to AC, we theorized that Wnt11 might have some general role in tumorigenesis. To investigate whether Wnt11 protein is present in ACs, AC samples were tested for Wnt11 protein expression. Primary non-cancerous control (Figure 1D) and AC (Figure 1E) tissues were stained for Wnt11 using immunohistochemistry. Wnt11 was detected in both normal and cancerous samples, although at higher levels in the tumours. The results emphasized that Wnt11 is necessary for the normal homeostasis of the tissue and its increased expression in the tumour correlates with the carcinogenic process. The relative nature of Wnt11 expression was further confirmed, when Wnt11 gene transcription was measured in an AC (A549) and an SCC (H157) cancer cell line. While Wnt11 levels were higher in both A549 and H157 than in normal, non-cancerous SAEC, several fold increase was detectable in Wnt11 mRNA expression in the AC (Figure 1F) than in the SCC cell line.

To find out more about the role of Wnt11 and potentially Wnt5a in the carcinogenic process, further studies were performed.

### Wnt11 and Wnt5a in NSCLC

To examine the role of non-canonical Wnts in LC development, first Wnt11 levels were modified in the A549 cell line using lentiviral transgenesis for overexpression and commercially available siWnt11 for transient suppression of Wnt11 (Figure S1).

**Figure 2:**
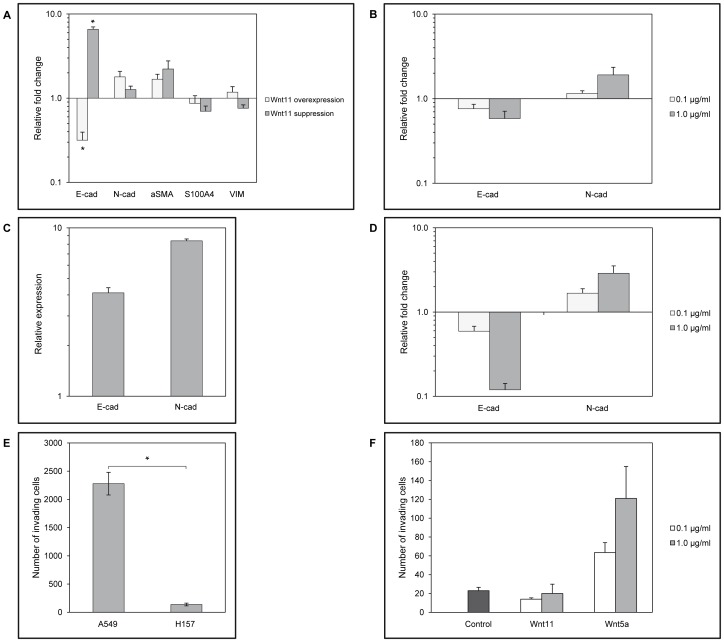
Effects of Wnt11 and Wnt5a in tumour development. **A:** Effects of Wnt11 overexpression and suppression in A549 AC cell line. Gene expression of A549-GFP and A549 treated with control siRNA served as reference, respectively. Note the decreased expression of E-cadherin in Wnt11-A549 (p<0.011), and the increased E-cadherin level following siWnt11 treatment (p<0.006). **B:** Recombinant Wnt11 treatment of SAEC cultures. Gene expression of untreated control cells was used as reference. Note the concentration dependent “cadherin switch” upon rWnt11 treatment. (Data are representative of five independent experiments where SAEC was used of five individual donors of different ages and sexes). **C:** Expression of E- and N-cadherin in primary adenocarcinoma lung tissue sample compared to its healthy autologous control pair. Data are representative of the analysis of three independent tissue pairs. **D:** Recombinant Wnt5a treatment of SAEC cultures. Gene expression of untreated control cells was used as reference. Note the concentration dependent “cadherin switch” upon rWnt5a treatment. (The presented data are representative of five independent experiments where SAEC was used as control of five individual donors of different ages and sexes). **E:** Invasion assay. The A549 AC cell line that expresses high levels of Wnt11 is more invasive than the H157 SCC cell line with lower expression of Wnt11 (p<0.008). **F:** Invasion assay. Primary non-cancerous SAEC were treated with 1 µg/ml rWnt11 and rWnt5a for three days prior to the assay. Only the rWnt5a treated SAEC migrated faster than the non-treated control cells. (The results are representative of three independent experiments where SAEC was used from three individual donors of different ages).

As in a limited number of previous studies Wnt11 has been implicated as a regulator of EMT (Zhang, JBC, 2012), several EMT molecular markers including S100, aSMA, and VIM were measured. Only E- and N-cadherin mRNA levels changed significantly in Wnt11 overexpressing-A549 cells (Figure 2A), drastically decreasing E- and increasing N-cadherin expression. In contrast, transient inhibition of Wnt11 translation using siWnt11 could only increase E-cadherin mRNA transcription while N-cadherin levels remained unchanged implicating E-cadherin as a Wnt11 target (Figure 2A). To test whether Wnt11 has a similar effect in primary pulmonary epithelium, SAEC-s presenting mixed epithelial phenotypes (Figure S2) were treated with different concentrations (0.1 and 1.0 µg/ml) of purified recombinant Wnt11. The experiments provided further evidence that Wnt11 regulates cadherin levels (Figure 2B) as E-cadherin expression decreased, while N-cadherin mRNA levels increased in Wnt11 treated SAEC-s in a concentration dependent manner. To investigate E- and N-cadherin expression in AC-s where Wnt11 protein levels are higher than in their non-cancerous autologous controls (Figure 1D and E), qRT-PCR analysis was performed. In the Wnt11 enriched environment of AC-s, both E- and N-cadherin levels were higher than in the non-cancerous control tissues (Figure 2C) indicating Wnt11 as a regulator of cadherin expression and emphasizing the regulatory role of additional factors present in the molecular microenvironment of the tissue. Based on the literature [22] Wnt5a and Wnt11 can support or even replace one another *in vivo* while controlling tissue development and functions, therefore the effect of Wnt5a was also tested on E- and N-cadherin expression. Similarly to rWnt11, rWnt5a (0.1 and 1.0 µg/ml) was able to down-regulate E- and up-regulate N-cadherin expression in primary SAEC (Figure 2D).

To investigate whether added Wnt11 or Wnt5a can modulate E- and N-cadherin expression in LC cell lines, both A549 and H157 cell lines were exposed to rWnt5a and Wnt11 at the concentration of 0.1 and 1.0 µg/ml. Added rWnt5a to AC cell line was able to further decrease E-cadherin, and had no significant effect on N-cadherin, while rWnt5a had no significant effect on either genes in the H157 cell line. Added rWnt11 in the H157 SCC cell line increased N-cadherin expression and had no effect on the already non-detectable E-cadherin levels (Figure S3).

As current literature concerning the role of Wnt5a and Wnt11 in cadherin regulation [23], [24] and therefore in cellular motility is contradicting, an invasion assay was performed, where non-treated primary SAEC, rWnt5a and rWnt11 treated SAEC as well as A549 AC and H157 SCC cells were tested (Figure 2E, 2F). In the invasion assay the A549 cell line that expresses Wnt11 at high levels moved much faster than the H157 cell line that highly expresses Wnt5a (Figure 2E). In contrast, rWnt5a treated non-cancerous SAEC were more invasive than the rWnt11 treated SAEC which were not moving faster than the controls (Figure 2F), indicating that primary, non-cancerous cells might require additional signals or longer exposure to Wnt11 to achieve increased motility.

### Inhibition of Canonical Wnt Signalling and β-catenin Localization

In the TaqMan array of SCC samples, apart from up-regulation of non-canonical Wnt-s, suppression of canonical Wnt signalling was also detected that might have an important role in modulating the tissue’s microenvironment.

**Figure 3:**
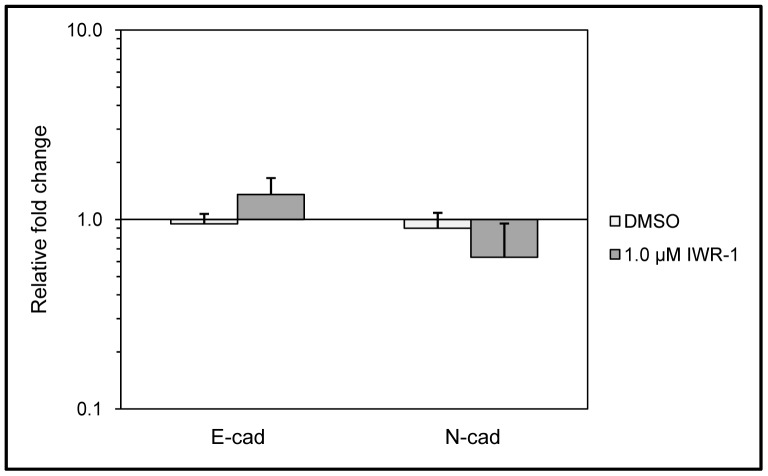
Effects of β-catenin inhibition on cadherin gene expression. Suppression of canonical Wnt signalling in SAEC using 1 µg/ml IWR-1 inhibitor or DMSO as diluent control. Gene expression of non-treated SAEC was used as reference. Note the increased E-cadherin and decreased N-cadherin mRNA expressions. (The results are representative of three independent experiments where SAEC was used from three individual donors of different ages).

As the Wnt11 promoter contains two conserved Tcf/LEF binding sites and two conserved GATA sites, canonical Wnt signals and GATA family members can regulate Wnt11 transcription directly. In contrast, the Wnt5a promoter region harbours numerous *cis*-acting elements including several GC boxes and Sp1, AP1, and AP2 binding motifs [25]. To test how β-catenin activity affects Wnt11 and Wnt5a expression as well as cadherin mRNA levels, cells were treated with a β-catenin inhibitor, IWR-1 (1.0 µg/ml) for 24 hours. Inhibition of β-catenin function resulted in increased expression of E- and reduced N-cadherin expression (Figure 3), while both Wnt11 and Wnt5a transcription was slightly reduced (data not shown).

**Figure 4:**
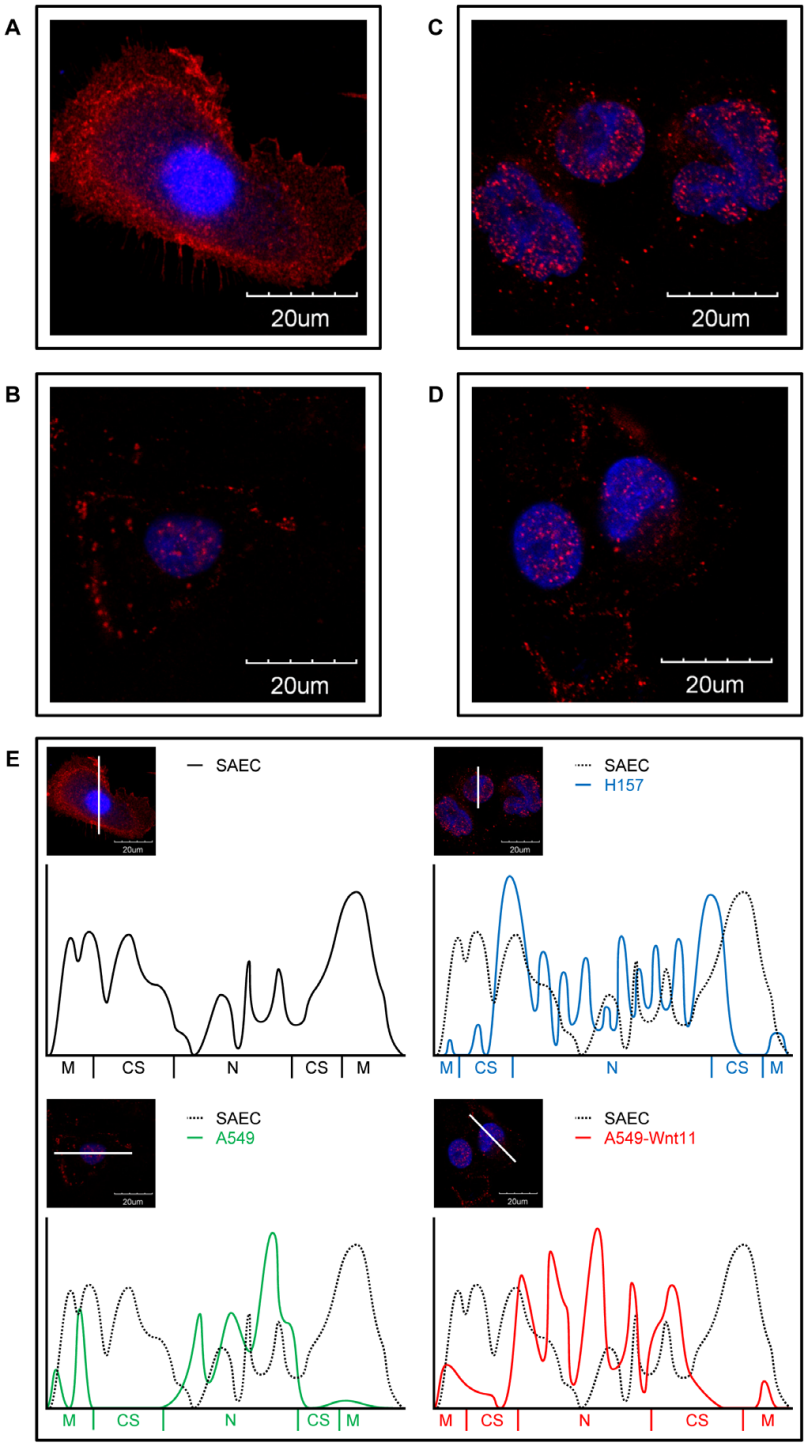
Localization of β-catenin. A: Immunofluorescent staining of SAEC. **B**: Immunofluorescent staining of normal A549. **C**: Immunofluorescent staining of Wnt11 overexpressing A549. **D**: Immunofluorescent staining of H157 monolayer cell cultures. (60x image, red: β-catenin, blue: DAPI). Note the dramatic increase in nuclear localization and the decrease in cellular membrane localization of A549 AC, Wnt11-A549 and H157 SCC cell lines compared to the normal pulmonary epithelium (SAEC). Data presented are representative of three independent experiments. **E**: Densitometry of immunofluorescent images of SAEC, A549, Wnt-11-A549 and H157 cells. Note the increased nuclear localization of β-catenin particularly in the Wnt11-A549 cell line. (M: cellular membrane, CS: cytosol, N: nucleus).

Additionally, β-catenin is not only important in the regulation of gene transcription but also in cellular adhesion. β-catenin associates with the cytoplasmic domain of cadherin and directly links to the actin cytoskeleton through α-catenin in a dynamic cadherin adhesion complex. Localization of β-catenin protein therefore was investigated in primary, non-cancerous SAEC (Figure 4A), A549 AC cell line (Figure 4B), Wnt11-A549 (Figure 4C) and an SCC cell line, H157 (Figure 4D). Cells were stained for β-catenin using protein specific antibody and the nucleus with DAPI, then staining intensity was scanned in a cross-section of the cells (Figure 4E). In the normal pulmonary epithelium, SAEC, β-catenin staining was strong in the cellular membrane and not in the nucleus indicating that β-catenin acts primarily in cellular adhesion and much less of the protein is involved in forming a transcription complex. In contrast, dramatic increase was detected in nuclear localization of β-catenin in Wnt11-A549 AC and in the H157 SCC cell lines while no significant amount of β-catenin was detected in their cellular membranes indicating severely weakened cellular adhesion.

## Discussion

Malfunction of genes and pathways controlling developmental processes have long been studied in association with cancers. Malfunction of Wnt related events have frequently been singled out as one of the causes of carcinogenesis. While Wnts seem to be important in the carcinogenic process of LC-s also, the complex molecular background of mixed LC types has been notoriously difficult to decipher.

Recent studies, including our TaqMan analysis, confirmed a differential molecular background to NSCLC sub-types AC-s and SCC-s involving up-regulation of canonical signalling in AC-s [26] and increased non-canonical Wnt signalling in SCC-s. While these results should be sufficient enough to identify additional therapeutic targets that could aid development of effective therapies, the underlying molecular complexity of AC and SCC works against this goal. In the present study we aimed to provide further details to the molecular background of pulmonary carcinogenesis and to connect individually observed molecular alterations during the process.

Similarly to previous studies, our experiments detected a marked increase in Wnt5a levels in SCC. As Wnt5a is a regulator of fibroblast proliferation and resistance to apoptosis [27] increased tissue mass of tumours with high Wnt5a levels is understandable. Interestingly, the role of Wnt5a in carcinogenesis is still ambiguous, as it can exhibit tumour suppressor activities in some cancers, like thyroid, brain, breast and colorectal, but is aberrantly up-regulated and associated with tumour progression in cancers of the lung, stomach and prostate. Increased Wnt5a levels in SCC samples might also be significant as an inducer of LC, as cigarette smoke extracts [28] trigger Wnt5a expression in various types of exposed tissues including macrophages and cells of epithelial origin. Comparative analysis of gene expression in AC and SCC tumour tissues has additionally identified Wnt11 and its receptor Fzd-7 [29] as potential regulators of SCC development. However, while the increase in Wnt5a levels was specific to SCC, the Wnt11 increase was only relative to AC. Similarly to SCC, AC-s had also shown up-regulated Wnt11 levels indicating a general role for Wnt11 in the carcinogenic process. Our studies using primary pulmonary epithelial tissues as well as cell lines identified Wnt5a and Wnt11 as regulators of cadherin expression, most likely the “cadherin switch” that is a characteristic marker of tumour progression. Our studies also confirmed that the effects of both Wnt5a and Wnt11 are concentration dependent and presumably modified by additional microenvironmental signals. Perhaps even a combination of Wnt5a/Wnt11 in SCC and Wnt7b/Wnt11 in AC. The additional signals could also include for example different levels of Wnt11 receptor Fzd-7 in NSCLC sub-types that was detected in the AC and SCC samples (unpublished observations), as well as activators of e.g. the SP1, AP1 or AP2 transcription factors that could modify Wnt5a transcription. As Wnt5a can also activate gene transcription via AP1, SCC tumour development might be associated with an auto-regulatory loop of Wnt5a. According to the literature, Wnt5a dependent E-cadherin down-regulation, however, is independent of the canonical Wnt pathway in melanoma type skin cancer and favours the PKC dependent up-regulation of a transcriptional repressor, Snail [30]. Addition to modification of cadherin expression, both Wnt5a and Wnt11 adjusted β-catenin localization, redirecting β-catenin from cellular adhesion foci to the nucleus. The fact that Wnt11 can loosen cellular adhesion is not a novel concept. Previous studies have found that Wnt11 can promote ubiquitination and therefore degradation of the focal adhesion protein paxillin supporting turnover of focal adhesions which process is required for cell migration [31].

As an additional factor, a general down-regulation of canonical Wnt signalling was also detected in SCC. To investigate the potential significance of canonical Wnt pathway suppression, β-catenin was chemically inhibited in cell lines and in primary pulmonary epithelial cells, then Wnt5a and Wnt11, E-and N-cadherin expression was studied. While Wnt11 and Wnt5a levels were slightly suppressed upon β-catenin inhibition, E-cadherin was up- and N-cadherin was down-regulated. While the outcomes of the above experiments are in agreement with previous description of non-canonical Wnt signalling regulation, it doesn’t provide explanation for our results of primary tissues where up-regulation of non-canonical Wnts with parallel down-regulation of the canonical Wnt pathway was detected. Although further studies are essential, our observations in primary tumours are not unique in lung SCC, as a similar molecular pattern was detected in non-melanoma type, squamous cell skin cancer [32] also.

While the main molecular trends in AC and especially in SCC development are apparent, gene expression changes that are currently unexplained might hold the key to effective therapeutic design. Unfortunately, detailed analysis of all identified molecular alterations has gone way beyond the scope of the present study one can still hypothesise about important future directions of the investigation. In contrast to SCC for example, development of AC is orchestrated by de-regulation of canonical Wnt pathway suppressors and up-regulation of Wnt7b, a lung epithelium-linked Wnt [33], [34]. It has also been determined that Wnt7b promoter activity is regulated by a homeo-domain transcription factor, TTF1 (Thyroid Transcription Factor 1) [33], responsible for alveolar epithelial development, indicating that mutation or malfunction of TTF1 might be responsible for AC-type carcinogenesis. Emphasizing the complex regulation of LC development, increased mRNA expression of Fzd-3, a natural receptor for Wnt5a [35] was also detected in AC-s, rather than in SCC although the significance of this finding is unclear. Meanwhile, Fzd-10, a receptor for Wnt7a [36]–[38] as well as Wnt7b [38] showed an increase in SCC. Whether Fzd-10 up-regulation is a sign of compensatory attempt for inhibited canonical Wnt signalling is awaiting further experimentation. Additionally, high levels of Wnt6 were also detected in SCC samples. Based on studies in human gastric cancer (GC) cells, WNT6 was able to increase resistance to apoptotic cell death induced by chemotherapeutic agents and enhance resistance of GC cells to anthracycline drugs [39]. Whether Wnt6 is involved in regulation of drug resistance in LC remains to be seen. Based on the summary of present findings one potential target in lung SCC therapy could involve AP1 induced gene transcription using the dominant negative c-jun as transcriptional repressor as in the transgenic TAM67 (dominant-negative c-Jun) mouse model both skin [40] and lung cancer tumour progression was successfully inhibited as a result of interference with Wnt5a signalling [41].

**Figure 5:**
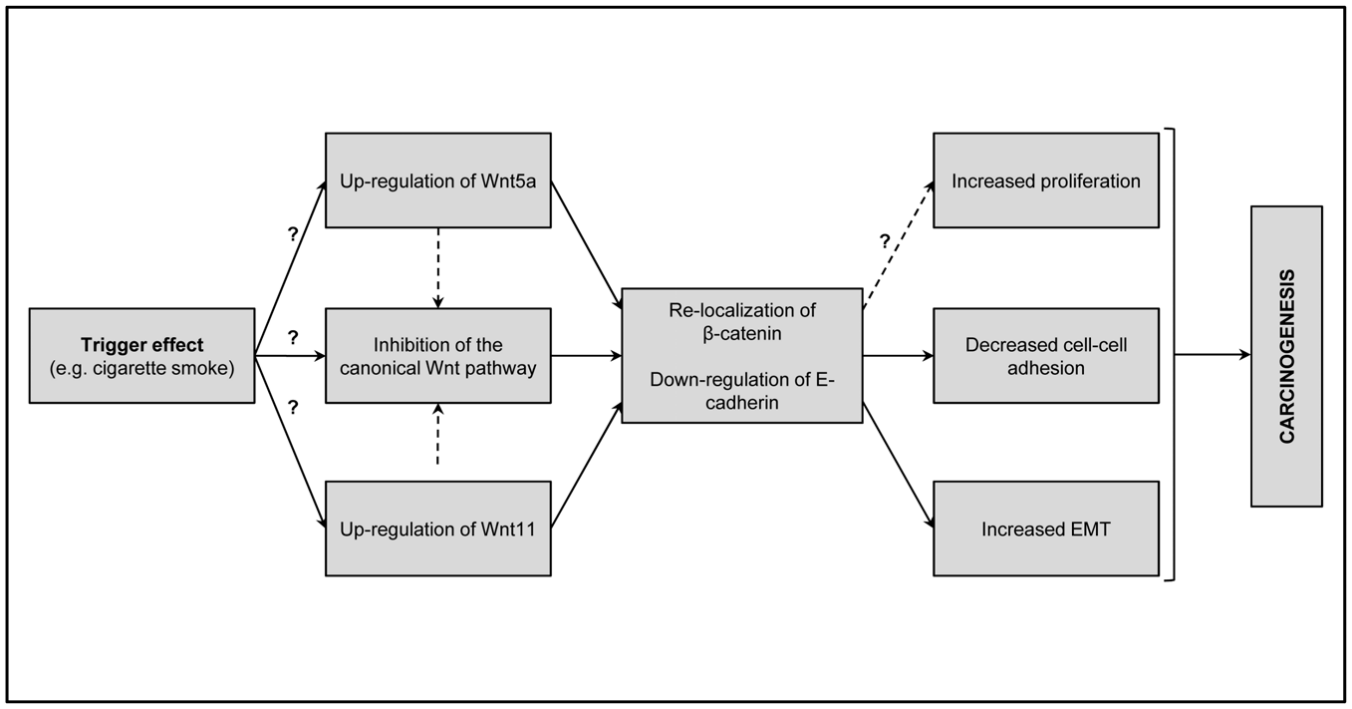
Predicted molecular pathway interactions during SCC development.

In summary (Figure 5) we theorize that cigarette smoke triggers up-regulation of Wnt5a that initiates proliferation and aids suppression of canonical Wnt signalling that leads to decreased participation of β-catenin in cellular adhesion. Increased Wnt11 ratio decreases cellular adhesion even further by suppressing E-cadherin expression, by reducing β-catenin molecules in anchorage function and by inducing degradation of the focal adhesion protein paxillin.

Although further studies are clearly required, by highlighting the overlapping function of molecular pathways of LC-s, our work has provided additional facts to aid better understanding of the molecular background of NSCLC sub-types which might help development of curative therapies in the future.

## Supporting Information

Figure S1 **Wnt11 overexpression and suppression in A549 cells.** A549 pulmonary adenocarcinoma cells were transfected with a lentiviral bicistonic construct encoding full-lenght Wnt11-IRES-GFP. Control A549 cells were transfected with GFP only. **A:** Quantitative RT-PCR measurement of Wnt11 mRNA levels in A549 and Wnt11-A549 cells. **B:** Detection of Wnt11 protein by Western blotting. A549 cells were lysed in SDS sample buffer containing 10% 2-ME and heated for 5 minutes at 95C. Then SDS-PAGE was performed and proteins were blotted onto nitrocellulose membrane. Membrane was blocked in TBS containing 2% BSA and 0.1% Tween 20. Rabbit anti-Wnt11 pAb (Abcam) and mouse anti-β-actin mAb (Sigma) primary antibodies were used. HRP labelled antibodies specific for rabbit and mouse IgG, respectively, were used as secondary reagents. Blots were visualized using the chemiluminescent Supersignal Kit (Pierce). **C**: transfection efficiency of cy3 labelled siRNA-s was monitored using fluorescence microscopy. **D**: Quantitative RT-PCR measurement of Wnt11 mRNA levels in control and siWnt11-A549 cells.
(TIF)

Figure S2 **Expression profile of various epithelial markers in SAECs.** mRNA levels of SAEC were measured with qRT-PCR for the following types of epithelial markers of commercially obtained SAEC: AQP3: Aquaporin 3 (NM_004925, AGCCCCTTCAGGATTTCCA-GACCCAAATTCCGGTTCCA, 86 bp); AQP4: Aquaporin 4 (NM_004028, GCGAGGACAGCTCCTATGAT-ACTGGTGCCAGCATGAATC, 110 bp); AQP5: Aquaporin 5 (NM_001651.2, CCCTGCGGTGGTCATGA-ATGGGCCCTACCCAGAAAAC, 60 bp); MUC-1: Mucin 1, cell surface associated (NM_001204286.1, CTCATTGCCTTGGCTGTCTGT-GATGTCCAGCTGCCCGTAGT, 57 bp); FOXJ1: Forkhead box protein J1 (NM_001454, CGAGGCACTTTGATGAAGC-CAACTTCTGCTACTTCCGCC, 110 bp); CC10: Clara cell 10 (NM_003357.4, CGAGGCACTTTGATGAAGC-CAACTTCTGCTACTTCCGCC, 135 bp). Relative β-actin was used as the normalizer gene (SD from duplicate readings). The results indicated a mixed phenotype of SAECs. The present figure is a representative of three separate tests.
(TIF)

Figure S3 **rWnt treatment of A549 and H157 cell lines. A:** rWnt5a treatment of the A549 AC cell line. Note the decreased expression of E-cadherin and the slight increase in N-cadherin expression. **B:** rWnt11 treatment of the H157 SCC cell line. Note the lack of E-cadherin and increased N-cadherin expression. **C:** rWnt5a treatment of the H157 SCC cell line. Note the lack of E-cadherin and increased N-cadherin expression.
(TIF)

Table S1 Comparative analysis of primary lung adenocarcinoma (AC) samples to their tissue autologous controls.
(DOCX)

Table S2 Comparative analysis of primary lung squamous cell carcinoma (SCC) samples to their tissue autologous controls.
(DOCX)

Table S3 Comparative analysis of primary lung squamous cell carcinoma to adenocarcinoma samples.
(DOCX)

## References

[1] Parkin DM, Bray F, Ferlay J, Pisani P (2005) Global cancer statistics, 2002 Cancer J Clin. 55: 74–108. 10.3322/canjclin.55.2.74 15761078

[2] Pongracz JE, Stockley RA (2006) Wnt signaling in lung development and diseases. Respiratory Res 7: 15. 10.1186/1465-9921-7-15 PMC1397816 16438732

[3] Calvo R, West J, Franklin W, Erickson P, Bemis L, et al (2000) Altered HOX and WNT7A expression in human lung cancer. Proc Natl Acad Sci USA 97: 12776–12781. 11070089 10.1073/pnas.97.23.12776 PMC18840

[4] He B, You L, Uematsu K, Xu Z, Lee AY, et al (2004) A monoclonal antibody against Wnt-1 induces apoptosis in human cancer cells. Neoplasia 6: 7–14. 15068666 10.1016/s1476-5586(04)80048-4 PMC1508626

[5] You L, He B, Xu Z, Uematsu K, Mazieres J, et al (2004) Inhibition of Wnt-2-mediated signaling induces programmed cell death in non-small-cell lung cancer cell. Oncogene 23: 6170–6174. 15208662 10.1038/sj.onc.1207844

[6] Winn RA, Marek L, Han SY, Rodriguez K, Rodriguez N, et al (2005) Restoration of Wnt-7a expression reverses non-small cell lung cancer cellular transformation through frizzled-9-mediated growth inhibition and promotion of cell differentiation. J Biol Chem 280: 19625–19634. 15705594 10.1074/jbc.M409392200

[7] Uematsu K, He B, You L, Xu Z, McCormick F, et al (2003) Activation of the Wnt pathway in non small cell lung cancer: evidence of dishevelled overexpression. Oncogene 22: 7218–7221. 14562050 10.1038/sj.onc.1206817

[8] Nozaki I, Tsuji T, Iijima O, Ohmura Y, Andou A, et al (2001) Reduced expression of REIC/Dkk-3 gene in non-small cell lung cancer. Int J Oncol 19: 117–121. 11408931 10.3892/ijo.19.1.117

[9] Wissmann C, Wild PJ, Kaiser S, Roepcke S, Stoehr R, et al (2003) WIF1, a component of the Wnt pathway, is down-regulated in prostate, breast, lung, and bladder cancer. J Pathol 201: 204–212. 14517837 10.1002/path.1449

[10] Mazieres J, He B, You L, Xu Z, Lee AY, et al (2004) Wnt inhibitory factor-1 is silenced by promoter hypermethylation in human lung cancer. Cancer Res 64: 4717–4720. 15256437 10.1158/0008-5472.CAN-04-1389

[11] Lee AY, He B, You L, Dadfarmay S, Xu Z, et al (2004) Expression of the secreted frizzled-related protein gene family is downregulated in human mesothelioma. Oncogene 23: 6672–6676. 15221014 10.1038/sj.onc.1207881

[12] Taki M, Kamata N, Yokoyama K, Fujimoto R, Tsutsumi S, et al (2003) Down-regulation of Wnt-4 and up-regulation of Wnt-5a expression by epithelial-mesenchymal transition in human squamous carcinoma cells. Cancer Sci 94: 593–597. 12841867 10.1111/j.1349-7006.2003.tb01488.x PMC11160266

[13] Brabletz T, Hlubek F, Spaderna S, Schmalhofer O, Hiendlmeyer E, et al (2005) Invasion and metastasis in colorectal cancer: epithelial-mesenchymal transition, mesenchymal-epithelial transition, stem cells and beta-catenin. Cells Tissues Organs 179: 56–65. 15942193 10.1159/000084509

[14] Sunaga N, Kohno T, Kolligs FT, Fearon ER, Saito R, et al (2001) Constitutive activation of the Wnt signaling pathway by CTNNB1 (beta-catenin) mutations in a subset of human lung adenocarcinoma. Genes Chromosomes Cancer 30: 316–321. 11170292 10.1002/1098-2264(2000)9999:9999<::aid-gcc1097>3.0.co;2-9

[15] Ueda M, Gemmill RM, West J, Winn R, Sugita M, et al (2001) Mutations of the beta- and gamma-catenin genes are uncommon in human lung, breast, kidney, cervical and ovarian carcinomas. Br J Cancer 85: 64–68. 11437403 10.1054/bjoc.2001.1863 PMC2363927

[16] Blanco D, Vicent S, Elizegi E, Pino I, Fraga MF, et al (2004) Altered expression of adhesion molecules and epithelial-mesenchymal transition in silica-induced rat lung carcinogenesis. Lab Invest 84: 999–1012. 15195114 10.1038/labinvest.3700129

[17] Akiri G, Cherian MM, Vijayakumar S, Liu G, Bafico A, et al (2009) Wnt pathway aberrations including autocrine Wnt activation occur at high frequency in human non-small-cell lung carcinoma. Oncogene 28: 2163–2172. 19377513 10.1038/onc.2009.82 PMC4451819

[18] Hu Y, Galkin AV, Wu C, Reddy V, Su AI (2011) CAFET algorithm reveals Wnt/PCP signature in lung squamous cell carcinoma. PLoS One 6: e25807. 22016777 10.1371/journal.pone.0025807 PMC3189939

[19] Lockwood WW, Chari R, Coe BP, Thu KL, Garnis C, et al (2010) Integrative genomic analyses identify BRF2 as a novel lineage-specific oncogene in lung squamous cell carcinoma. PLoS Med 7: e1000315. 20668658 10.1371/journal.pmed.1000315 PMC2910599

[20] Bovia F, Salmon P, Matthes T, Kvell K, Nguyen TH, et al (2003) Efficient transduction of primary human B lymphocytes and nondividing myeloma B cells with HIV-1-derived lentiviral vectors. Blood 101: 1727–1733. 12406892 10.1182/blood-2001-12-0249

[21] Brambilla E, Travis WD, Colby TV, Corrin B, Shimosato Y (2001) The new World Health Organization classification of lung tumours. Eur Respir J 18: 1059–1068. 11829087 10.1183/09031936.01.00275301

[22] Brandon C, Eisenberg LM, Eisenberg CA (2000) WNTsignaling modulates the diversification of hematopoietic cells. Blood 96: 4132–4141. 11110684

[23] Medrek C, Landberg G, Andersson T, Leandersson K (2009) Wnt-5a-CKI{alpha} signaling promotes {beta}-catenin/E-cadherin complex formation and intercellular adhesion in human breast epithelial cells. J Biol Chem 284: 10968–10979. 19244247 10.1074/jbc.M804923200 PMC2667782

[24] Arnsdorf EJ, Tummala P, Jacobs CR (2009) Non-canonical Wnt signaling and N-cadherin related beta-catenin signaling play a role in mechanically induced osteogenic cell fate. PloS One 4: e5388. 19401766 10.1371/journal.pone.0005388 PMC2670536

[25] Danielson KG, Cohen IR, Huebner K, Nicholls JM, Iozzo RV (1995) Characterization of the complete genomic structure of the human WNT-5A gene, functional analysis of its promoter, chromosomal mapping, and expression in early human embryogenesis. J Biol Chem 270: 31225–31234. 8537388 10.1074/jbc.270.52.31225

[26] Nguyen DX, Chiang AC, Zhang XH-F, Kim JY, Kris MG, et al (2009) WNT/TCF signaling through LEF1and HOXB9 mediates lung adenocarcinoma metastasis. Cell 138: 1–12. 10.1016/j.cell.2009.04.030 PMC2742946 19576624

[27] Vuga LJ, Ben-Yehudah A, Kovkarova-Naumovski E, Oriss T, Gibson KF, et al (2009) WNT5A is a regulator of fibroblast proliferation and resistance to apoptosis. Am J Respir Cell Mol Biol 41: 583–589. 19251946 10.1165/rcmb.2008-0201OC PMC2778165

[28] Hussain M, Rao M, Humphries AE, Hong JA, Liu F, et al (2009) Tobacco Smoke Induces Polycomb-Mediated Repression of Dickkopf-1 in Lung Cancer Cells. Cancer Res 69: 3570. 19351856 10.1158/0008-5472.CAN-08-2807 PMC8374472

[29] Kim GH, Her JH, Han JK (2008) Ryk cooperates with Frizzled 7 to promote Wnt11-mediated endocytosis and is essential for Xenopus laevis convergent extension movements. J Cell Biol 182: 1073–1082. 18809723 10.1083/jcb.200710188 PMC2542470

[30] Dissanayake SK, Wade M, Johnson CE, O'Connell MP, Leotlela PD, et al (2007) The Wnt5A/protein kinase C pathway mediates motility in melanoma cells via the inhibition of metastasis suppressors and initiation of an epithelial to mesenchymal transition. J Biol Chem 282: 17259–17271. 17426020 10.1074/jbc.M700075200 PMC2263117

[31] Iioka H, Iemura S, Natsume T, Kinoshita N (2007) Wnt signalling regulates paxillin ubiquitination essential for mesodermal cell motility. Nat Cell Biol 9: 813–821. 17558393 10.1038/ncb1607

[32] Pourreyron C, Reilly L, Proby C, Panteleyev A, Fleming C, et al (2012) Wnt5a is strongly expressed at the leading edge in non-melanoma skin cancer, forming active gradients, while canonical Wnt signalling is repressed. PLoS One 7: e31827. 22384081 10.1371/journal.pone.0031827 PMC3285195

[33] Weidenfeld J, Shu W, Zhang I, Millar SE, Morrisey EE (2002) The Wnt7b promoter is regulated by TTF-1, GATA6, and Foxa2 in lung epithelium. J Biol Chem 277: 21061–21070. 11914369 10.1074/jbc.M111702200

[34] Shu W, Jiang YQ, Lu MM, Morrisey EE (2002) Wnt7b regulates mesenchymal proliferation and vascular development in the lung. Development 129: 4831–4842. 12361974 10.1242/dev.129.20.4831

[35] Kawasaki A, Torii K, Yamashita Y, Nishizawa K, Kanekura K, et al (2007) Wnt5a promotes adhesion of human dermal fibroblasts by triggering a phosphatidylinositol-3 kinase/Akt signal. Cell Signal 19: 2498–2506. 17804197 10.1016/j.cellsig.2007.07.023

[36] Carmon KS, Loose DS (2008) Secreted frizzled-related protein 4 regulates two Wnt7a signaling pathways and inhibits proliferation in endometrial cancer cells. Mol Cancer Res 6: 1017–1028. 18567805 10.1158/1541-7786.MCR-08-0039

[37] Kawakami Y, Wada N, Nishimatsu S, Nohno T (2000) Involvement of frizzled-10 in Wnt-7a signaling during chick limb development. Dev Growth Differ 42: 561–569. 11142678 10.1046/j.1440-169x.2000.00545.x

[38] Wang Z, Shu W, Lu MM, Morrisey EE (2005) Wnt7b activates canonical signaling in epithelial and vascular smooth muscle cells through interactions with Fzd1, Fzd10, and LRP5. Mol Cell Biol 25: 5022–5030. 15923619 10.1128/MCB.25.12.5022-5030.2005 PMC1140585

[39] Yuan G, Regel I, Lian F, Friedrich T, Hitkova I, et al (2013) WNT6 is a novel target gene of caveolin-1 promoting chemoresistance to epirubicin in human gastric cancer cells. Oncogene 32: 375–387. 22370641 10.1038/onc.2012.40

[40] Kang MI, Baker AR, Dextras CR, Cabarcas SM, Young MR, et al (2012) Targeting of Noncanonical Wnt5a Signaling by AP-1 Blocker Dominant-Negative Jun When It Inhibits Skin Carcinogenesis. Genes Cancer 3: 37–50. 22893789 10.1177/1947601912448820 PMC3415668

[41] Tichelaar JW, Yan Y, Tan Q, Wang Y, Estensen RD, et al (2010) A dominant-negative c-jun mutant inhibits lung carcinogenesis in mice. Cancer Prev Res 3: 1148–1156. 10.1158/1940-6207.CAPR-10-0023 PMC2933283 20716630

