# Supplementary figures and images for: Down-Regulation of Canonical and Up-Regulation of Non-Canonical Wnt Signalling in the Carcinogenic Process of Squamous Cell Lung Carcinoma
Source: PLoS One. 2013 Mar 7;8(3):e57393. doi: 10.1371/journal.pone.0057393 (PMC3591434; doi:10.1371/journal.pone.0057393)

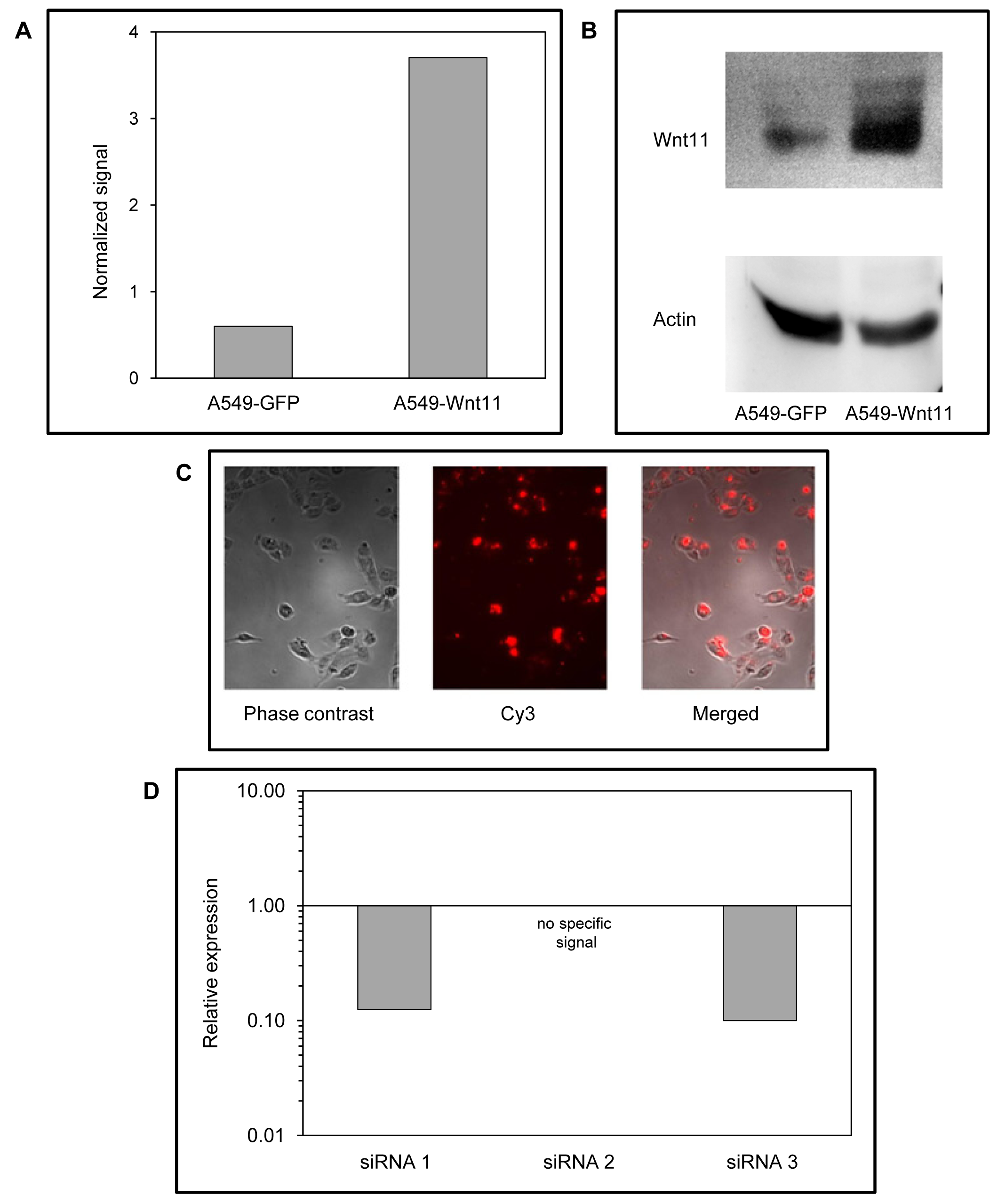

Supplement: Figure S1 — Wnt11 overexpression and suppression in A549 cells. A549 pulmonary adenocarcinoma cells were transfected with a lentiviral bicistonic construct encoding full-lenght Wnt11-IRES-GFP. Control A549 cells were transfected with GFP only. A: Quantitative RT-PCR measurement of Wnt11 mRNA levels in A549 and Wnt11-A549 cells. B: Detection of Wnt11 protein by Western blotting. A549 cells were lysed in SDS sample buffer containing 10% 2-ME and heated for 5 minutes at 95C. Then SDS-PAGE was performed and proteins were blotted onto nitrocellulose membrane. Membrane was blocked in TBS containing 2% BSA and 0.1% Tween 20. Rabbit anti-Wnt11 pAb (Abcam) and mouse anti-β-actin mAb (Sigma) primary antibodies were used. HRP labelled antibodies specific for rabbit and mouse IgG, respectively, were used as secondary reagents. Blots were visualized using the chemiluminescent Supersignal Kit (Pierce). C: transfection efficiency of cy3 labelled siRNA-s was monitored using fluorescence microscopy. D: Quantitative RT-PCR measurement of Wnt11 mRNA levels in control and siWnt11-A549 cells. (TIF) [file pone.0057393.s001.tif]

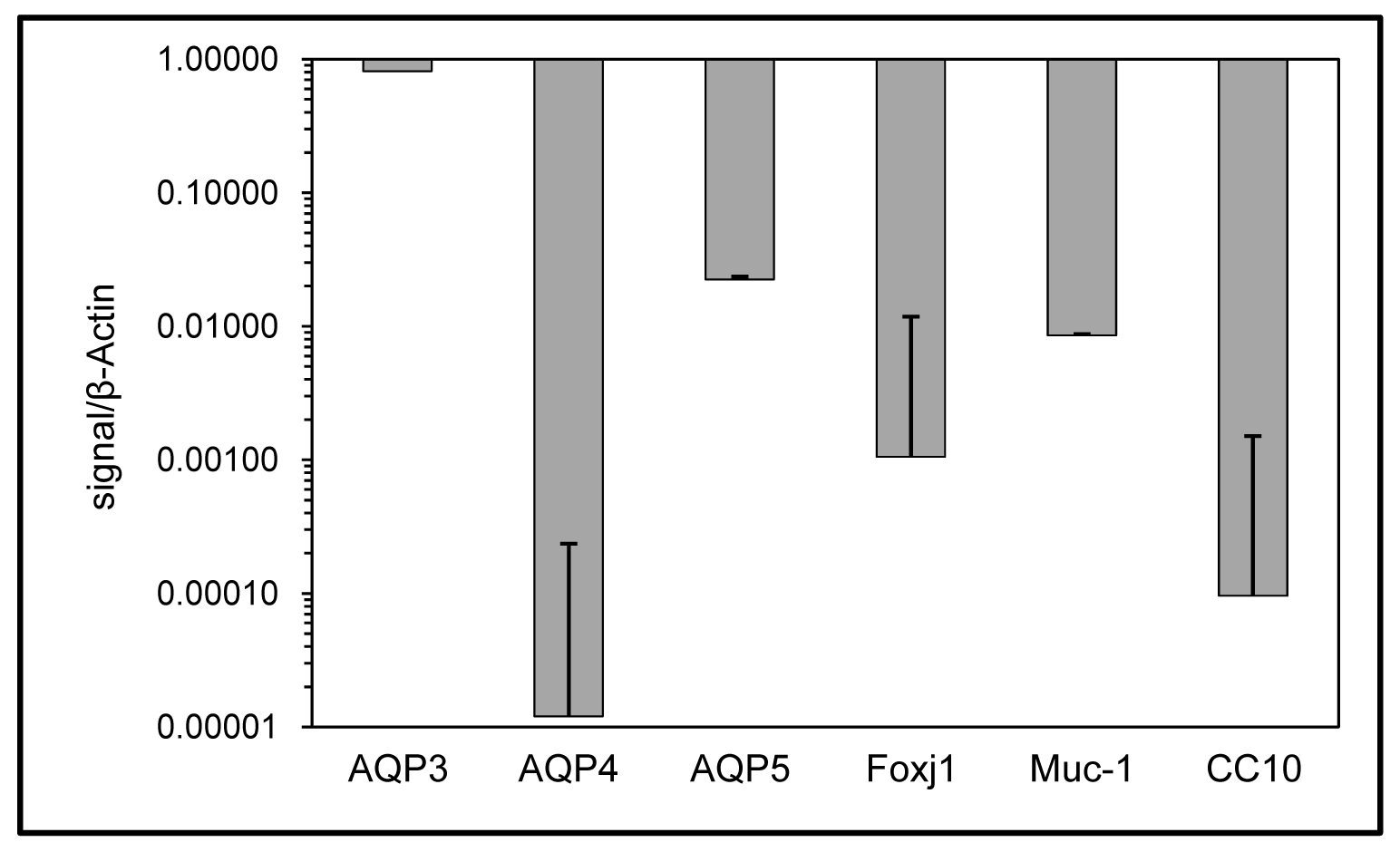

Supplement: Figure S2 — Expression profile of various epithelial markers in SAECs. mRNA levels of SAEC were measured with qRT-PCR for the following types of epithelial markers of commercially obtained SAEC: AQP3: Aquaporin 3 (NM_004925, AGCCCCTTCAGGATTTCCA-GACCCAAATTCCGGTTCCA, 86 bp); AQP4: Aquaporin 4 (NM_004028, GCGAGGACAGCTCCTATGAT-ACTGGTGCCAGCATGAATC, 110 bp); AQP5: Aquaporin 5 (NM_001651.2, CCCTGCGGTGGTCATGA-ATGGGCCCTACCCAGAAAAC, 60 bp); MUC-1: Mucin 1, cell surface associated (NM_001204286.1, CTCATTGCCTTGGCTGTCTGT-GATGTCCAGCTGCCCGTAGT, 57 bp); FOXJ1: Forkhead box protein J1 (NM_001454, CGAGGCACTTTGATGAAGC-CAACTTCTGCTACTTCCGCC, 110 bp); CC10: Clara cell 10 (NM_003357.4, CGAGGCACTTTGATGAAGC-CAACTTCTGCTACTTCCGCC, 135 bp). Relative β-actin was used as the normalizer gene (SD from duplicate readings). The results indicated a mixed phenotype of SAECs. The present figure is a representative of three separate tests. (TIF) [file pone.0057393.s002.tif]

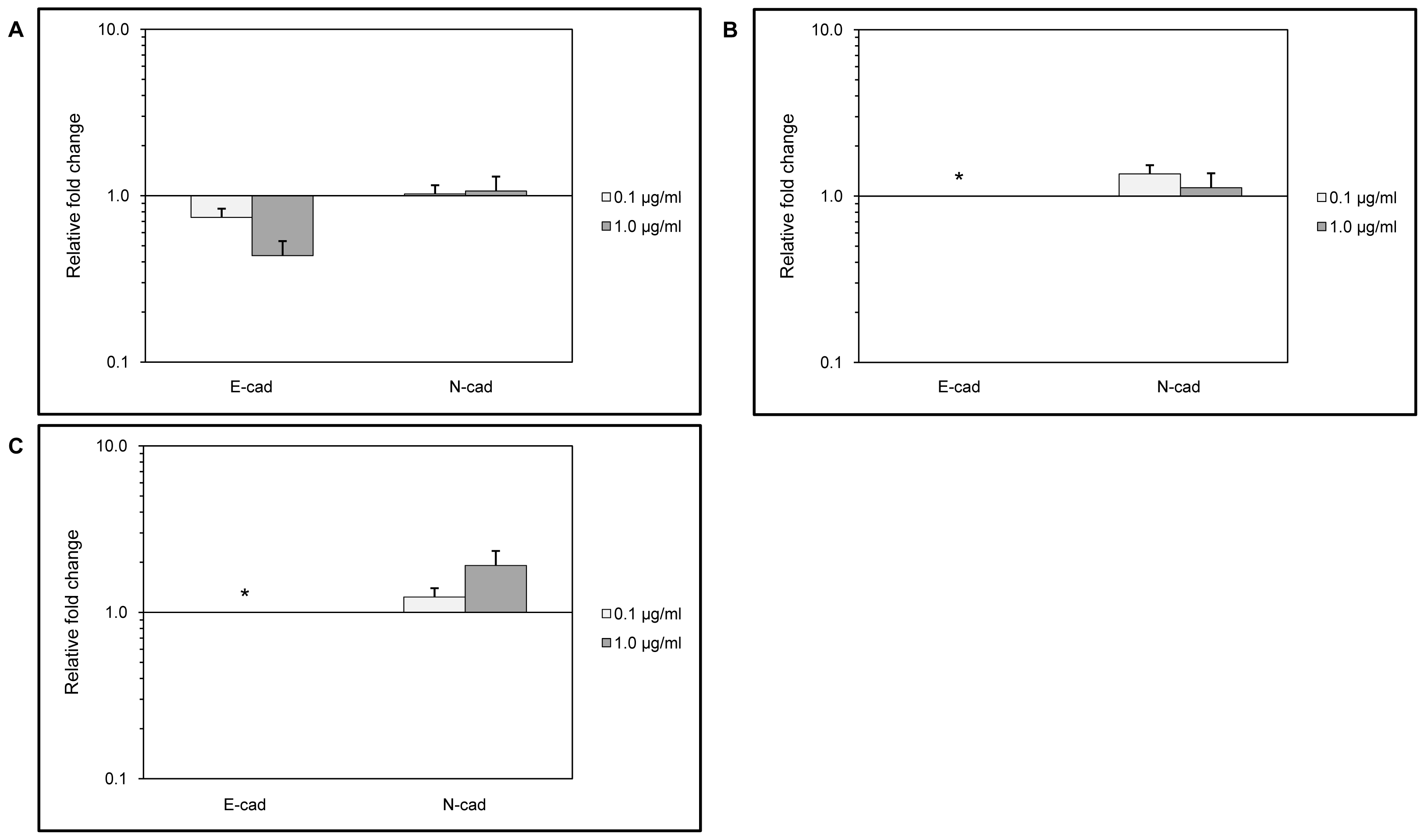

Supplement: Figure S3 — rWnt treatment of A549 and H157 cell lines. A: rWnt5a treatment of the A549 AC cell line. Note the decreased expression of E-cadherin and the slight increase in N-cadherin expression. B: rWnt11 treatment of the H157 SCC cell line. Note the lack of E-cadherin and increased N-cadherin expression. C: rWnt5a treatment of the H157 SCC cell line. Note the lack of E-cadherin and increased N-cadherin expression. (TIF) [file pone.0057393.s003.tif]
