# Supplementary material for: Down-Regulation of Canonical and Up-Regulation of Non-Canonical Wnt Signalling in the Carcinogenic Process of Squamous Cell Lung Carcinoma
Source: PLoS One. 2013 Mar 7;8(3):e57393. doi: 10.1371/journal.pone.0057393 (PMC3591434; doi:10.1371/journal.pone.0057393)
Supplement: Table S1 — Comparative analysis of primary lung adenocarcinoma (AC) samples to their tissue autologous controls. (DOCX) [file pone.0057393.s004.docx]

**Table S1.** **Comparative analysis of primary lung adenocarcinoma (AC) samples to their tissue autologous controls.**

| **Assay ID** | **Gene Symbol** | **Relative quantity** |
| --- | --- | --- |
| Hs00181051_m1 | APC | 0.22 |
| Hs00394718_m1 | AXIN-1 | 0.41 |
| Hs00610344_m1 | AXIN-2 | 0.09 |
| Hs00182707_m1 | BTRC | 0.31 |
| Hs00360360_m1 | CBY-1 | 0.48 |
| Hs00793391_m1 | CSNK1A1 | 0.52 |
| Hs00371196_m1 | CSNK1D | 0.51 |
| Hs00222804_m1 | CSNK1G1 | 0.47 |
| Hs00176258_m1 | CSNK1G2 | 0.29 |
| Hs00177858_m1 | CSNK1G3 | 0.18 |
| Hs00751002_s1 | CSNK2A1 | 0.55 |
| Hs00176505_m1 | CSNK2A2 | 0.33 |
| Hs00365835_m1 | CSNK2B | 0.50 |
| Hs00170025_m1 | CTNNB1 | 0.54 |
| Hs00172016_m1 | CTNNBIP1 | 0.38 |
| Hs00228693_m1 | CXXC4 | 0.66 |
| Hs00420410_m1 | DACT1 | 0.14 |
| Hs00183740_m1 | DKK-1 | 1.09 |
| Hs00205294_m1 | DKK-2 | 0.10 |
| Hs00247429_m1 | DKK-3 | 0.09 |
| Hs00746889_s1 | DVL-1 | 0.36 |
| Hs00182901_m1 | DVL-2 | 0.38 |
| Hs00610263_m1 | DVL-3 | 0.63 |
| Hs00914223_m1 | EP300 | 0.31 |
| Hs00606870_m1 | FBXW11 | 0.26 |
| Hs00271384_s1 | FRAT1 | 0.16 |
| Hs00362616_s1 | FRAT2 | 0.24 |
| Hs00173503_m1 | FRZB | 0.08 |
| Hs00268943_s1 | FZD-1 | 0.13 |
| Hs00361432_s1 | FZD-2 | 0.17 |
| Hs00184043_m1 | FZD-3 | 1.29 |
| Hs00201853_m1 | FZD-4 | 0.09 |
| Hs00171574_m1 | FZD-6 | 0.39 |
| Hs00275833_s1 | FZD-7 | 0.12 |
| Hs00259040_s1 | FZD-8 | 0.90 |
| Hs00268954_s1 | FZD-9 | 0.37 |
| Hs00273077_s1 | FZD-10 | 0.07 |
| Hs00219856_m1 | GSK3A | 0.50 |
| Hs00275656_m1 | GSK3B | 0.31 |
| Hs00230750_m1 | KREMEN-1 | 0.41 |
| Hs00212390_m1 | LEF-1 | 0.29 |
| Hs00182031_m1 | LRP-5 | 0.28 |
| Hs00233935_m1 | LRP-6 | 0.26 |
| Hs00153408_m1 | MYC | 0.40 |
| Hs00263894 | NKD-1 | 0.09 |
| Hs00212076_m1 | NLK | 0.37 |
| Hs00224508_m1 | PORCN | 0.33 |
| Hs00427259_m1 | PPP2CA | 0.54 |
| Hs00204426_m1 | PPP2R1A | 0.50 |
| Hs00396159_m1 | PYGO1 | 0.17 |
| Hs00540767_m1 | PYGO2 | 0.43 |
| Hs00221873_m1 | RHOU | 0.38 |
| Hs00222121_m1 | SENP2 | 0.54 |
| Hs00610060_m1 | SFRP-1 | 0.15 |
| Hs00293258_m1 | SFRP-2 | 0.69 |
| Hs00180066_m1 | SFRP-4 | 0.20 |
| Hs00169366_m1 | SFRP-5 | 0.11 |
| Hs00188594_m1 | SLC9A3R | 1.39 |
| Hs00175273_m1 | TCF7 | 0.47 |
| Hs00229841_m1 | TCF7L1 | 0.32 |
| Hs00181036_m1 | TCF7L2 | 0.17 |
| Hs00270768_m1 | TLE1 | 0.24 |
| Hs00610086_m1 | TLE2 | 0.19 |
| Hs00183222_m1 | TLE3 | 0.29 |
| Hs00419101_m1 | TLE4 | 0.19 |
| Hs00226999_m1 | TLE6 | 0.33 |
| Hs00183662_m1 | WIF-1 | 0.03 |
| Hs00365573_m1 | WISP-1 | 1.08 |
| Hs00608224_m1 | WNT-2 | 0.08 |
| Hs00257131_m1 | WNT-2B | 0.12 |
| Hs00263977_m1 | WNT-3A | 0.03 |
| Hs00229142_m1 | WNT-4 | 0.47 |
| Hs00180103_m1 | WNT-5A | 0.29 |
| Hs00364142_m1 | WNT-5B | 0.43 |
| Hs00362452_m1 | WNT-6 | 0.47 |
| Hs00171699_m1 | WNT-7A | 0.04 |
| Hs00536497_m1 | WNT-7B | 2.13 |
| Hs00243321_m1 | WNT-9A | 0.07 |
| Hs00287409_m1 | WNT-9B | 0.50 |
| Hs00228741_m1 | WNT-10A | 0.33 |
| Hs00559664_m1 | WNT-10B | 0.12 |
| Hs00182986_m1 | WNT-11 | 0.07 |
| Hs00365138_m1 | WNT-16 | 0.36 |
